# Efficient Production of Vigorous Scions by Optimizing Leaf Retention in *Passiflora edulis*

**DOI:** 10.3390/plants14162483

**Published:** 2025-08-10

**Authors:** Xiuqing Wei, Yajun Tang, Jianglong Lai, Liang Li, Ping Zhou, Dong Yu, Limei Tang, Jiahui Xu

**Affiliations:** Fruit Research Institute, Fujian Academy of Agricultural Sciences, Fuzhou 350013, China; weixiuqing@faas.cn (X.W.); tangyajun@faas.cn (Y.T.);

**Keywords:** passion fruit, scion, leaf retention, axillary bud, auxin

## Abstract

*Passiflora edulis* propagation relies extensively on grafting, yet the optimization of pruning strategies for scion quality remains empirically guided. This study elucidates the physiological and molecular mechanisms underlying scion quality across five leaf retention treatments (0%, 25%, 50%, 75%, and unpruned control). The 50% partial leaf retention (50% PLR) treatment optimally promoted axillary bud development in passion fruit through coordinated physiological and molecular adaptations. This treatment significantly outperformed other treatments in terms of both bud sprouting rate and growth parameters (including length and diameter). Physiological analyses demonstrated transient auxin accumulation coupled with synchronized antioxidant system activation, maintaining redox homeostasis. Transcriptomic profiling identified upregulation of genes in the auxin signaling pathway and cytokinin activators, while dormancy-related genes were suppressed. These findings establish 50% PLR as an optimal threshold that balances photosynthetic capacity with hormonal regulation, providing a science-based strategy to standardize grafted seedling production, while enhancing scion quality for grafting efficiency.

## 1. Introduction

Passion fruit (*Passiflora edulis*) is a high-value tropical crop cultivated across many countries, with significant global production and economic importance [1,2]. As a perennial vine requiring intensive management, its commercial propagation predominantly relies on grafted seedlings to combat soil-borne pathogens like *Fusarium oxysporum* f. sp. *passiflorae*, which reduce yield and fruit quality [3]. However, the efficiency of grafting in passion fruit remains suboptimal, with success rates typically ranging between 60 and 75% in commercial nurseries [4,5]. A critical factor limiting grafting success is the inconsistent quality of scion material, particularly the physiological state of axillary buds used for propagation. Citrus (*Citrus aurantifolia*) cultivation provides a compelling case study, where systematic retention of rootstock leaves has been demonstrated to improve grafting success rates, primarily by maintaining adequate carbohydrate supply during the delicate graft union formation phase [6]. Similarly, in passion fruit stock plants (scion-donor plants), optimal leaf retention ensures hormonal homeostasis and sustained photoassimilate supply to developing axillary buds, directly addressing the scion quality inconsistency described earlier. Therefore, establishing standardized, science-based protocols for passion fruit propagation has become critically important for enhancing both operational efficiency and production consistency in commercial nurseries.

The success of passion fruit grafting hinges on stringent quality control for both rootstocks and scions. For rootstock preparation, dedicated seedlings are cultivated until stems reach 3–4 mm in diameter, then processed to 12–18 cm in height with complete defoliation to optimize graft union formation. These specifications, coupled with vigorous mother plants for scion production, collectively determine propagation efficiency and subsequent orchard performance. Crucially, the yield and quality of scions are intrinsically linked to the growth vigor of the mother plants used for scion collection, necessitating equally rigorous selection criteria for scion-donor stock. Current research on passion fruit production, domestically and internationally, has predominantly focused on rootstock disease resistance [3,5,7], while studies addressing the systematic management of stock plants for sustainable scion production remain notably scarce. The physiological basis for optimal scion preparation in passion fruit remains poorly understood, particularly regarding how leaf retention influences bud development and subsequent grafting performance. Current nursery practices employ arbitrary levels of leaf retention, typically ranging from 30 to 70% of the total leaf area, without scientific justification. This knowledge gap becomes particularly significant when considering the complex physiological responses of passion fruit to pruning. The species exhibits remarkably high photosynthetic rates, yet its leaves are highly susceptible to photooxidative damage when defoliated. Similar physiological trade-offs have been observed in grapevine (*Vitis vinifera* L.), where partial leaf removal in the range of 40–60% has been demonstrated to optimize budbreak by carefully balancing the maintenance of photosynthetic capacity with a reduction in apical dominance [8]. These findings from other species suggest that passion fruit may similarly respond to precisely calibrated leaf retention strategies, though species-specific studies are lacking.

Previous research in perennial crops has demonstrated that partial leaf retention promotes bud outgrowth through phytohormonal regulation [9], though passion fruit’s distinct physiology may involve specialized control mechanisms. The growth of axillary buds is fundamentally governed by the dynamic balance between auxin and cytokinin accumulation in dormant meristems. In apple, studies on the dwarfing rootstock M.9 have revealed its capacity to regulate scion growth by altering auxin transport efficiency and reducing the supply of root-derived cytokinins (particularly zeatin riboside, ZR) and gibberellins (GA_19_) to scions [10,11]. This hormonal modulation not only inhibits excessive vegetative growth but also promotes axillary bud differentiation. Importantly, pruning-induced leaf removal further disrupts apical dominance (*Malus spectabilis*) by triggering cytokinin signaling while suppressing auxin transport (NPA inhibition) and modifying strigolactone biosynthesis through MAX1/D14 gene regulation [12]. Parallel research in grapevine has demonstrated that retained leaves serve dual functions: they provide essential carbohydrate substrates while simultaneously synthesizing and transporting zeatin riboside directly to axillary buds [13]. These cross-species observations collectively highlight how strategic leaf area retention can physiochemically reprogram hormonal fluxes to control bud growth competence, though the specific implementation of these mechanisms in passion fruit remains to be elucidated.

Strategic pruning of stock plants is a pivotal determinant of plant growth, quality, and productivity in cultivation systems [14,15]. Through a multi-level investigation integrating five leaf retention treatments with physiological profiling and transcriptomic sequencing, we systematically elucidate how partial defoliation regulates axillary bud development at molecular and organismal levels. Our work not only establishes the first evidence-based leaf retention threshold (50% PLR) for passion fruit propagation but also deciphers its underlying phytohormonal crosstalk and metabolic reprogramming mechanisms. These findings establish an optimized and standardized protocol for sustaining scion quality in *Passiflora edulis*, ensuring consistent performance of grafted propagules.

## 2. Results

### 2.1. Effects of Differential Leaf Retention on Axillary Bud Development in P. edulis

**Figure 1:**
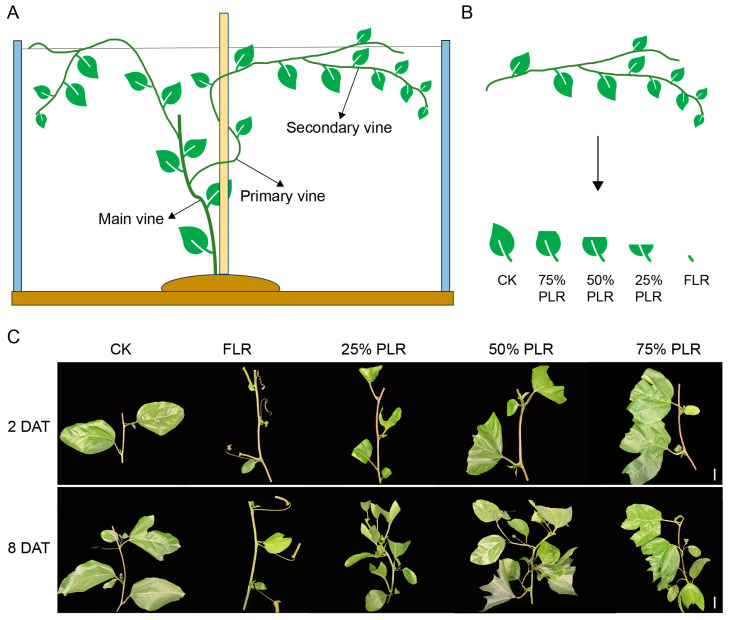
Axillary bud outgrowth under different leaf treatments of scions in passion fruit. (**A**) Morphology of passion fruit vine. (**B**) Different leaf treatments of scions in passion fruit. (**C**) Phenotypes of axillary buds at different time points (2 DAT and 8 DAT) after leaf treatments. DAT, days after leaf treatment.

*Passiflora edulis* exhibits a characteristic hierarchical vine architecture comprising three distinct orders of branching (Figure 1A). The primary vine (main stem) grows vertically from the base, requiring manual topping at 1.5–2 m height to promote lateral branching. Secondary vines emerge from primary vine leaf axils, forming the plant’s structural framework, with optimal nutrient allocation achieved by retaining 2–3 vigorous secondary vines per plant. Tertiary vines, developing from secondary vines, function as the principal fruit-bearing and cutting-propagating shoots, where their growth vigor and spatial density directly determine the yield potential and cutting quality. This tripartite architecture maintains vegetative–reproductive balance through apical dominance regulation.

**Figure 2:**
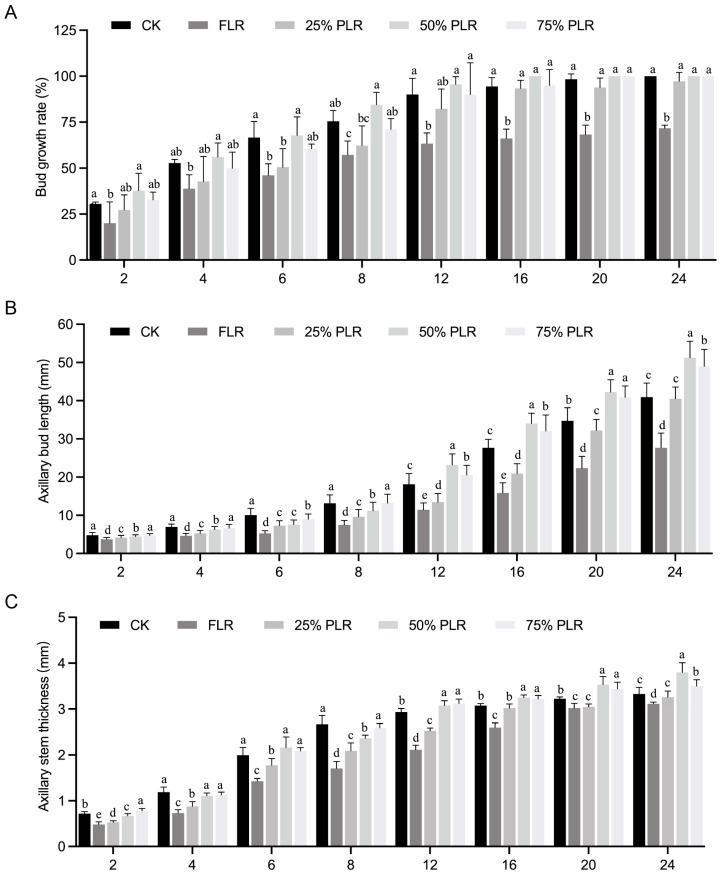
Axillary bud growth under different leaf pruning treatments (2-24 DAT). (**A**) Changes in axillary bud growth rate under different leaf pruning treatments. (**B**) Changes in axillary bud length with different treatments. (**C**) Changes in axillary stem thickness with different treatments. Letter in figure indicates significant differences between groups (*P* < 0.05, one-way ANOVA, Tukey’s HSD post hoc test).

In *Passiflora edulis* seedling propagation through grafting, secondary vines are routinely employed as the primary propagation material, making their structural and physiological characteristics critically important for determining the quality of the resultant seedlings. As the key structural determinant of mother plants, secondary vines significantly influence the cutting quality of tertiary vines through their pruning chronology and physiological status. In commercial propagation systems, standard practice involves regulated defoliation of secondary vines in stock plants to stimulate axillary bud outgrowth (developing tertiary vines). When tertiary vines reach the optimal 4 cm length, 6–10 cm stem segments containing these shoots are excised for use as grafting scions. To investigate these relationships, five distinct leaf retention treatments were applied to secondary vines: a control group (CK, with intact leaves), full leaf removal (FLR), and partial leaf retention at 25% (25% PLR), 50% (50% PLR), and 75% (75% PLR) per leaf (Figure 1B). Significant variations in axillary bud growth were observed across treatments at both 2 days after treatment (2 DAT) and 8 DAT (Figure 1C). The sprouting dynamics displayed treatment-specific patterns, with the sprouting rate following a biphasic trend characterized by an initial rapid growth phase (2-8 DAT) subsequently transitioning to asymptotic stabilization. Significant treatment differences were detected during early observation (*p* < 0.05). The 50% PLR exhibited the fastest bud sprouting rate, achieving complete sprouting by 16 DAT), followed by 75% PLR at 20 DAT and CK at 24 DAT (Figure 2A). In contrast, FLR exhibited the poorest performance, underscoring the crucial role of leaf retention in bud break.

Axillary bud growth metrics revealed distinct stratification among the treatments. By 24 DAT, the 50% PLR treatment produced significantly longer buds (51.26 ± 4.31 mm) compared to other treatments, whereas FLR yielded the shortest buds (27.71 ± 3.87 mm) (Figure 2B). Intermediate treatments (25% PLR and CK) showed comparable growth patterns (40.50 ± 3.06 mm vs. 40.93 ± 3.68 mm) (Figure 2B), suggesting a threshold effect of leaf retention on bud elongation. Diameter growth followed a biphasic development pattern, with greater expansion occurring during 2-8 DAT compared to 12-24 DAT across all treatments (Figure 2C). Specifically, the 50% PLR treatment achieved the maximum diameter (3.80 ± 0.21 mm) by 24 DAT, while FLR, despite its minimal baseline growth, exhibited the highest relative increase in diameter (Figure 2C). This observation implies the activation of compensatory growth mechanisms under conditions of severe leaf removal stress.

### 2.2. Oxidative Stress Response to Differential Leaf Retention in Axillary Buds

**Figure 3:**
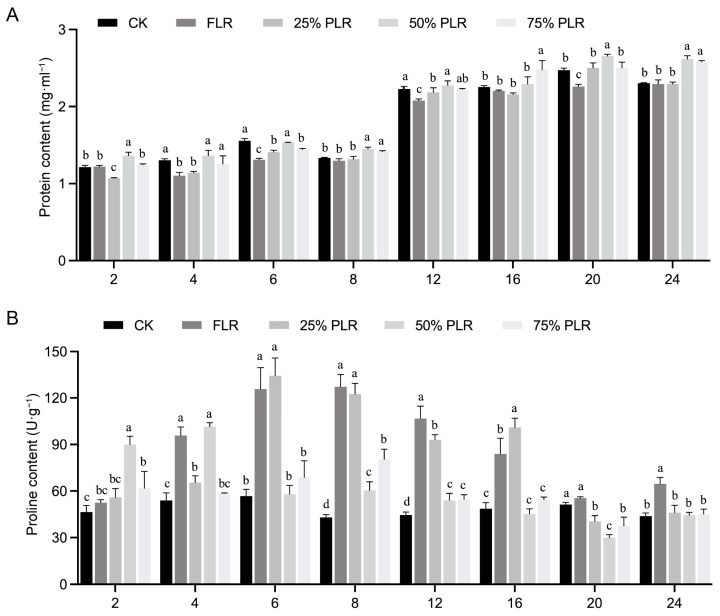
Dynamic changes in protein and proline contents in axillary buds under different leaf pruning treatments. Changes in protein content (**A**) and proline content (**B**) in axillary buds under different treatments. Letter in figure indicates significant differences between groups (*P* < 0.05, one-way ANOVA, Tukey’s HSD post hoc test).

The protein content dynamics revealed treatment-dependent stress responses. The control (CK) treatment maintained relatively stable protein levels with minimal temporal fluctuations, while significant variations were observed among the leaf retention treatment groups (Figure 3A). These differential protein accumulation patterns suggest that varying degrees of leaf retention exert distinct regulatory effects on metabolic processes in developing axillary buds. Proline (Pro) accumulation exhibited similar but more pronounced temporal patterns compared to the total protein content. All treatments showed rapid Pro accumulation during the initial 2-8 DAT, with peak concentrations occurring at either 6 or 8 DAT. Notably, the 25% PLR treatment reached its maximum Pro concentration (134.30 ± 11.58 U·g^−1^) at 6 DAT, while FLR demonstrated delayed peak accumulation at 8 DAT (127.29 ± 7.82 U·g^−1^) (Figure 3B), indicating differential timing in stress response activation. The control group maintained consistently lower Pro levels throughout the experimental period. Following the peak accumulation period, Pro contents gradually declined across all treatments, stabilizing near baseline levels by 24 DAT, suggesting progressive physiological acclimation to the treatment conditions.

**Figure 4:**
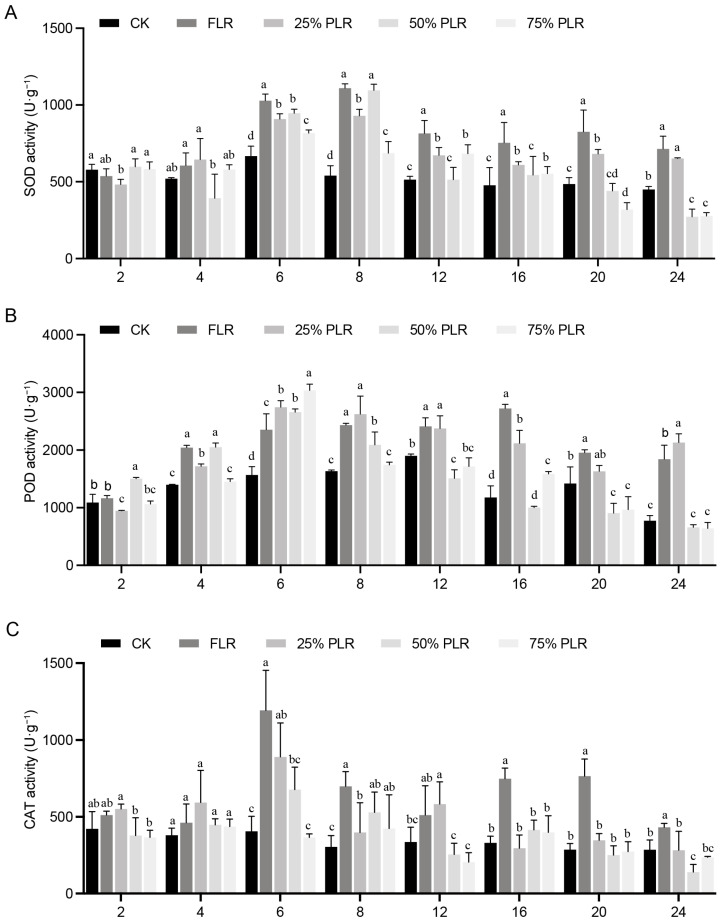
Dynamic changes in SOD, POD, and CAT activities in axillary buds under different leaf pruning treatments. Changes in SOD activity (**A**), POD activity (**B**), and CAT activity (**C**) in axillary buds under different treatments. Letter in figure indicates significant differences between groups (*P* < 0.05, one-way ANOVA, Tukey’s HSD post hoc test).

Antioxidant enzyme activities displayed treatment-specific temporal patterns that reflected oxidative stress responses. Superoxide dismutase (SOD) activity showed a progressive increase during the initial 8 DAT, peaking between 6 and 8 DAT (Figure 4A). The 50% PLR treatment exhibited significantly higher maximum SOD activity (1095.16 ± 39.65 U·g^−1^, *p* < 0.05) compared to other treatments. From 8 to 16 DAT, most treatments showed declining SOD activity with notable fluctuations, ultimately approaching baseline values by 24 DAT. Peroxidase (POD) activity demonstrated a rapid increase during the 2–8-day period, with the 75% PLR treatment reaching the highest peak activity at 6 DAT (3036.37 ± 107.89 U·g^−1^, *p* < 0.05) (Figure 4B). Following this initial phase, POD activity exhibited fluctuating patterns until 16 DAT, after which a consistent decline led to stabilization by 24 DAT. Catalase (CAT) activity showed similar dynamics, with all treatments reaching peak activity at 6 DAT. The FLR treatment induced significantly higher CAT activity (*p* < 0.05) compared to other treatments at this time point, indicating particularly strong oxidative stress under complete leaf removal conditions (Figure 4C). Post-peak, CAT activity gradually declined across all treatments, stabilizing near baseline levels by 24 DAT.

The coordinated temporal patterns of these biochemical markers demonstrate that all antioxidant enzymes returned to baseline levels by 24 DAT, confirming the transient nature of the oxidative stress response. The differential enzyme activation patterns among treatments suggest that moderate leaf retention (50–75%) provides an optimal balance between stress protection and growth maintenance in *P. edulis* propagation systems. The observed biochemical responses correlate well with the previously described morphological changes in axillary bud development [16], providing a comprehensive understanding of the physiological mechanisms underlying treatment effects.

### 2.3. Hormonal Dynamics Response to Differential Leaf Retention in Axillary Buds

**Figure 5:**
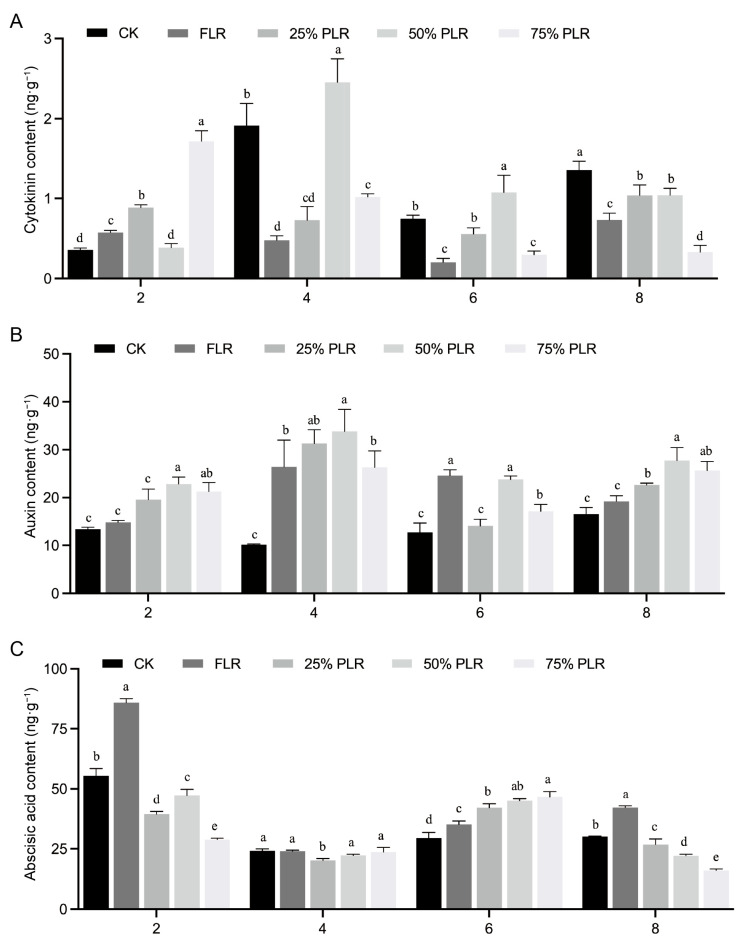
Dynamic changes in cytokinin, auxin, and abscisic acid contents in axillary buds under different leaf pruning treatments (2-8 DAT). Changes in cytokinin content (**A**), auxin content (**B**), and abscisic acid content (**C**) in axillary buds under different treatments. Letter in figure indicates significant differences between groups (*P* < 0.05, one-way ANOVA, Tukey’s HSD post hoc test).

A comprehensive analysis of agronomic traits, enzymatic activities, and redox metabolites in axillary buds revealed significant dynamic fluctuations across multiple parameters during the 2-8 DAT period. Given that axillary bud development is coordinately regulated by multiple phytohormones [17], we performed targeted hormone extraction and quantification from 2-8 DAT samples to elucidate the underlying regulatory mechanisms governing bud growth dynamics. All treatment groups displayed peak cytokinin (CTK) concentrations at 4 DAT, with particularly marked fluctuations observed in the 50% PLR and control (CK) treatments between 2 and 4 DAT. The 50% PLR treatment demonstrated a remarkable six-fold increase in CTK content, escalating from 0.39 ± 0.05 ng·g^−1^ fresh weight (FW) at 2 DAT to 2.46 ± 0.29 ng·g^−1^ FW at 4 DAT (*p* < 0.05) (Figure 5A), representing the highest concentration among all experimental groups during this critical period. In contrast, both the FLR and 25% PLR treatments showed minimal CTK variation throughout the observation window, maintaining consistently depressed levels that were significantly lower than those for other treatments during the 4-6 DAT phase.

Regarding auxin dynamics, experimental treatments consistently maintained higher indole-3-acetic acid (IAA) concentrations compared to the control across all sampling intervals. Initial measurements at 2 DAT revealed minimal IAA accumulation in both the CK and FLR treatments, which were statistically indistinguishable. The partial leaf retention treatments (25%, 50%, and 75% PLR) showed substantially elevated IAA levels, with the 50% PLR group displaying the most pronounced accumulation. Treatment differences reached maximal divergence at 4 DAT, coinciding with peak IAA biosynthesis, where the 50% PLR treatment achieved the maximal concentration (33.78 ± 4.64 ng·g^−1^ FW) (Figure 5B), representing a 2.7-fold increase over control values. This was followed by a moderate decline in most treatments by 6 DAT, though absolute levels remained elevated compared to those at baseline. By 8 DAT, treatment differences attenuated, showing convergent trends toward stabilization. Throughout the experimental period, the control maintained stable IAA concentrations (ranging from 10.13 ± 0.17 to 16.57 ± 1.36 ng·g^−1^ FW), indicating minimal endogenous fluctuation in the absence of treatment effects.

Abscisic acid (ABA) contents exhibited significant treatment-dependent variations across all four sampling timepoints. At 2 DAT, the FLR group showed the highest ABA concentration (85.89 ± 1.66 ng·g^−1^), significantly exceeding those of all other treatments. Substantial differences were also observed among the remaining groups, with the control showing marginally higher ABA levels compared to the 25% PLR and 50% PLR treatments (Figure 5C). During the subsequent 4–8 DAT period, ABA concentrations stabilized across all treatments, demonstrating attenuated fluctuation patterns and a gradual physiological adjustment to the imposed stress conditions. The coordinated temporal patterns of these phytohormones provide crucial insights into the complex regulatory networks governing axillary bud development under varying leaf retention regimes.

### 2.4. Phytohormonal Regulatory Mechanisms Underlying Axillary Bud Outgrowth

**Figure 6:**
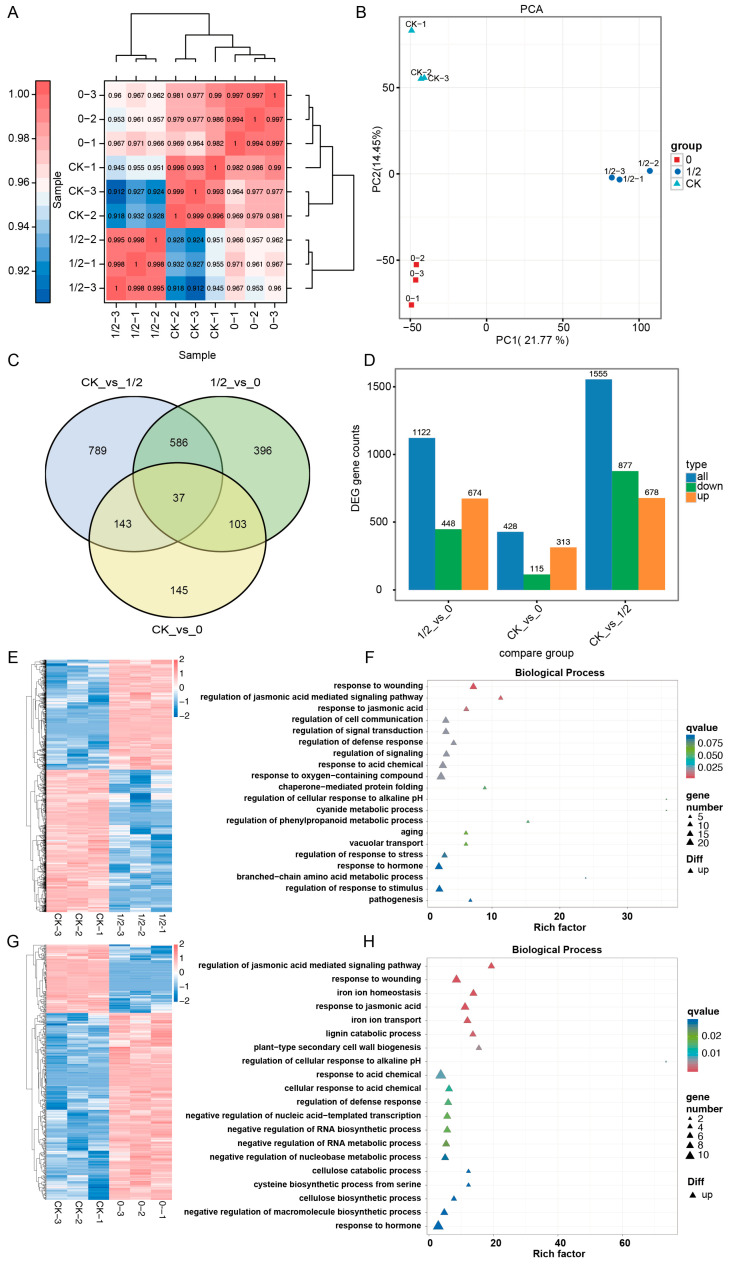
Transcriptomic profiling and comparative analysis of differentially expressed genes. (**A**) Correlation analysis of the three group samples. (**B**) PCA plots of the three group samples. (**C**) Venn diagram analysis of the differential gene set. (**D**) DEG statistics in three comparison groups. (**E**) Hierarchical cluster analysis of DEGs in CK_vs_1/2 comparison group. (**F**) Enriched biological process GO terms in CK_vs_1/2 comparison group. (**G**) Hierarchical cluster analysis of DEGs in CK_vs_0 comparison group. (**H**) Enriched biological process GO terms in CK_vs_0 comparison group.

Transcriptomic profiling revealed distinct differential gene expression patterns among the leaf retention treatments, generating approximately 274 million high-quality reads (Table S1). Correlation analysis demonstrated clear separation between the three experimental groups CK, 50% PLR, and FLR (Figure 6A). Principal component analysis (PCA) further confirmed minimal intra-group variation alongside significant inter-group differentiation (Figure 6B). Comparative analysis identified the fewest differentially expressed genes (DEGs) in the CK and FLR (CK_vs_0) comparison, with 428 total DEGs (313 upregulated and 115 downregulated) (Figure 6C,D, Table S4). In contrast, the CK and 50% PLR (CK_vs_1/2) comparison exhibited the most extensive transcriptional changes, with 1555 DEGs (877 upregulated and 678 downregulated), while the 50% PLR and FLR (1/2_vs_0) comparison showed intermediate changes (1122 DEGs: 674 upregulated, 448 downregulated) (Figure 6D). Gene ontology analysis revealed that the DEGs were predominantly associated with cellular oxygen responses, intracellular components, and signaling regulation in cellular components. Biological processes were dominated by hormone responses in CK_vs_50% PLR (Figure 6E,F) and CK_vs_FLR (Figure 6G,H), whereas metabolic processes, cellular regulation, and localization were enriched in 50% PLR_vs_0 (Figure S1).

Treatment-specific GO enrichment patterns demonstrated distinct functional specializations. The CK_vs_FLR DEGs showed pronounced enrichment in biological processes related to wound response, jasmonic acid signaling, and iron ion homeostasis; cellular components including the apoplast and cell wall; and molecular functions such as cytochrome binding and ferric ion binding (Table S2). The CK_vs_50% PLR comparison exhibited unique enrichment in carbohydrate metabolism, membrane constituents, and endopeptidase inhibitor/O-glycosyl hydrolase activities. In contrast, DEGs from the 50% PLR_vs_FLR comparison showed distinct enrichment profiles, with biological processes predominantly associated with cell wall organization, carbohydrate metabolism, and cellulose biosynthesis; cellular components involving membrane protein complexes; and molecular functions including O-glycosyl hydrolase activity and carbohydrate binding.

KEGG pathway analysis of the top 20 enriched pathways highlighted treatment-specific metabolic reprogramming. The CK_vs_FLR comparison showed dominant pathways in α-linolenic acid and cyanoamino acid metabolism, with upregulated genes particularly enriched in cyanoamino acid metabolism and glycosaminoglycan degradation (Table S3). The CK_vs_50% PLR comparison exhibited prominent involvement of endoplasmic reticulum protein processing, MAPK signaling, and cyanoamino acid metabolism, with upregulated genes enriched in benzoxazinoid biosynthesis and downregulated genes in N-glycan and steroid biosynthesis. The 50% PLR_vs_FLR comparison featured cyanoamino acid metabolism and endoplasmic reticulum protein processing as the dominant pathways, with upregulated genes showing enrichment in N-glycan flavonoid biosynthesis.

**Figure 7:**
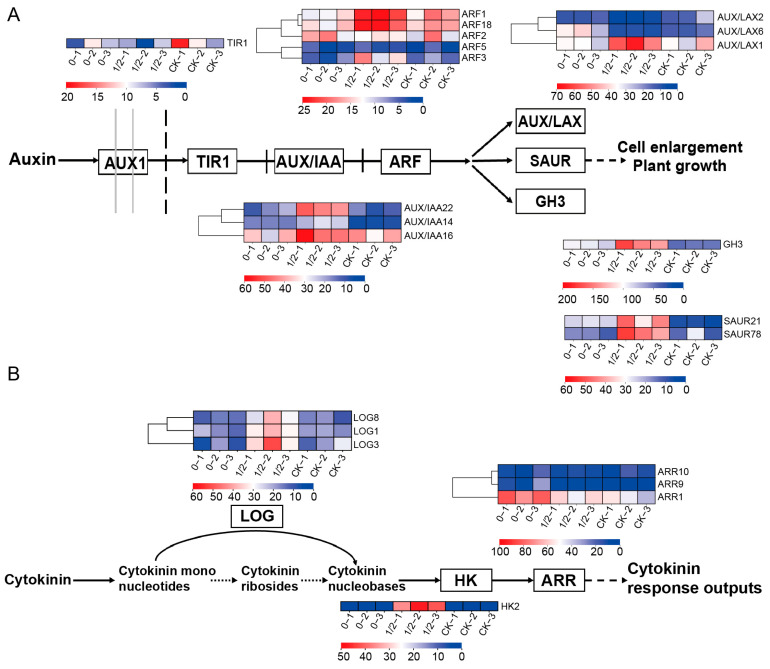
Expression profiles of hormone-related differentially expressed genes under different treatments. Key enzymes involved in auxin signaling pathway (**A**) and cytokinin signaling pathway (**B**).

Integrated analysis indicated that the DEGs were primarily distributed in metabolic pathways, suggesting that axillary buds respond to defoliation stress through accelerated carbohydrate synthesis and amino acid metabolism. The 50% PLR treatment appeared to coordinate growth promotion via synergistic regulation of amino acid and sugar metabolism genes. Given the observed auxin content variations, cluster analysis identified key auxin-associated DEGs including dormancy regulators (DRM1/ARP), signaling components (AUX/IAA16 and AUX/IAA22), transporters (AUX/LAX1), and auxin response factors (ARF1 and ARF18), along with auxin-responsive genes *GH3*, *SAUR21*, and *SAUR78* showing significantly elevated expression in 50% PLR compared to both the CK and FLR treatments (Figure 7A). Parallel analysis of cytokinin signaling revealed the cytokinin biosynthesis gene *LOG1* (*LONELY GUY 1*), its homolog *LOG3*, the response regulator *ARR1*, and histidine kinase *HK2* as central components mediating cytokinin responses (Figure 7B). These coordinated expression patterns demonstrate that the identified genes collectively participate in both auxin- and cytokinin-mediated developmental processes, including cell differentiation, stress adaptation, and bud outgrowth regulation, thereby establishing their pivotal role in governing axillary bud responses to varying leaf retention conditions.

**Figure 8:**
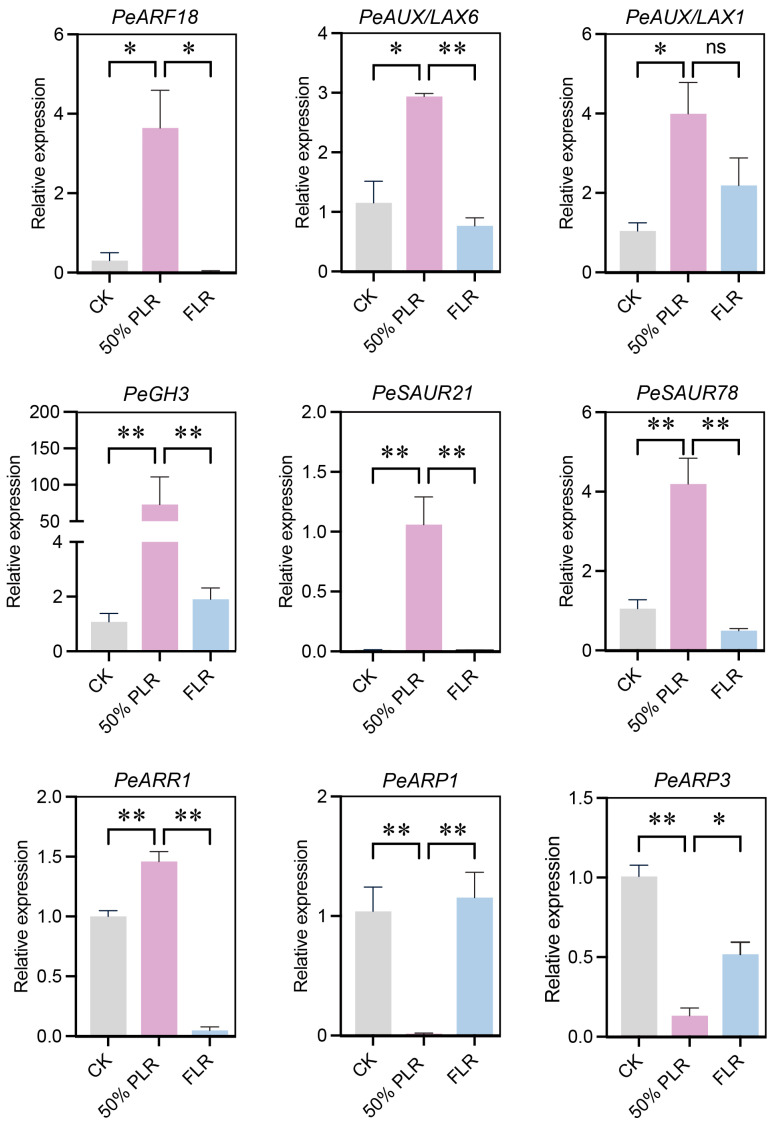
Expression patterns of significantly expressed genes in scion leaves with different retention levels. Statistically significant differences were determined by Student’s *t*-test: ns, no significant difference, * *P* < 0.05, ** *P* < 0.01.

qRT-PCR validation in scion leaves confirmed the RNA-seq expression patterns of selected genes. *PeARF18*, *PeAUX/LAX6*, *PeAUX/LAX1*, *PeGH3*, *PeSAUR21*, *PeSAUR78*, and *PeARR1* showed significant upregulation in 50% PLR, while *PeARP1* and *PeARP3* (dormancy/auxin repressor proteins) were downregulated, which is consistent with auxin and cytokinin’s synergistic promotion of axillary bud growth (Figure 8). These findings provide comprehensive molecular insights into axillary bud responses to differential leaf retention treatments, revealing coordinated phytohormone signaling and metabolic reprogramming underlying bud outgrowth regulation.

## 3. Discussion

Our multi-omics investigation revealed that 50% partial leaf retention (PLR) promotes axillary bud outgrowth in Passiflora edulis through a sophisticated integration of antioxidant defense, metabolic reprogramming, and hormonal crosstalk. The treatment’s effectiveness stems from its dual capacity to (i) maintain redox homeostasis through coordinated upregulation of SOD, POD, and CAT genes, correlating with observed enzyme activity peaks during 6-8 DAT, and (ii) activate the MAPK signaling pathway, which likely amplifies stress-responsive transcription factors while preventing oxidative damage. This balanced response contrasts sharply with complete defoliation (FLR), which induced a jasmonate-dominated defense program, a finding consistent with Cheng et al.’s report of JA-mediated growth inhibition in defoliated woody perennials [18]. Our metabolic profiling further demonstrated that 50% PLR preferentially enhances cellulose biosynthesis (supporting cell wall loosening) and benzoxazinoid production (enhancing stress tolerance), while FLR redirects carbon flux toward α-linolenic acid metabolism, effectively prioritizing defense over growth.

The hormonal regulation patterns provide mechanistic clarity for these phenotypic outcomes. The 50% PLR treatment established an optimal auxin–cytokinin balance through three coordinated actions: (i) upregulating AUX/LAX influx carriers (LAX1, LAX6) and ARF response factors (ARF1, ARF18) to enhance auxin signaling competency, (ii) activating cytokinin biosynthesis via LOG1 and signal transduction through ARR1, and (iii) modulating cellular expansion via SAUR21/78 while suppressing dormancy-imposing DRM1/ARP genes. This tripartite regulation creates a permissive environment for bud break by simultaneously promoting mitotic activity (through cytokinin) and cell wall remodeling (via auxin-mediated SAURs). Notably, the FLR treatment’s contrasting hormonal profile, which features GH3-mediated IAA inactivation and sustained DRM1 expression, effectively maintains bud dormancy, corroborating recent models of auxin–cytokinin antagonism in axillary meristems [19].

At the systems level, 50% PLR achieves an exceptional equilibrium between source–sink dynamics and signaling networks. The treatment’s unique transcriptional signature, which combines enhanced carbohydrate metabolism with endoplasmic reticulum protein processing, suggests efficient resource allocation where photosynthetic products simultaneously fuel growth and stress adaptation. This metabolic flexibility likely underlies the treatment’s superior performance, as it avoids the energy trade-offs characteristic of FLR’s defense-oriented response. From an applied perspective, our findings validate 50% PLR as a science-based protocol for passion fruit nurseries, where it can improve grafting efficiency by maintaining optimal donor plant physiology. The principles uncovered, particularly the 50% threshold for leaf retention and its effects on phytohormone–metabolic network coordination, may inform propagation strategies for other perennial crops facing similar source–sink challenges during vegetative propagation.

## 4. Materials and Methods

### 4.1. Plant Materials and Growth Conditions

Passion fruit plants were grown in a greenhouse in Zhangzhou, Fujian Province, China. Plant materials were selected from scion mother plants of the *Passiflora edulis* ‘Qinmi’ cultivar cultivated under greenhouse conditions (25 °C). The selection criteria required plants exhibiting uniform growth patterns and normal physiological development, with plants demonstrating comparable phenological stages and the absence of visible pathological symptoms or nutritional deficiencies. The plant growth substrate consisted of a mixture of peat moss, coconut coir, and perlite in a 5:4:1 ratio (*v*/*v*/*v*).

### 4.2. Measurements

Secondary vines of *Passiflora edulis* mother plants with uniform length, diameter, and nodal position were selected and labeled. For each stock plant, 1–2 secondary vines from the apical, median, and basal regions were selected. A total of 20 secondary vines were selected per treatment with three biological replicates. Six axillary buds below the apex of each secondary vine were monitored. The bud sprouting rate (%) was calculated as the proportion of sprouted buds to total observed buds. Vernier calipers (precision: 0.01 mm) were used to measure bud length and diameter at 2–4-day intervals throughout the experimental period.

### 4.3. Plant Hormone Components Assay

Plant hormone analysis was conducted according to a previous study [20]. Axillary bud sampling was initiated at 2 days post-leaf pruning and repeated at 2–4-day intervals. For each replicate per treatment, 3–4 buds were randomly collected and pooled. Samples were immediately snap-frozen in liquid nitrogen and stored at −80 °C until analysis. Frozen buds were lyophilized and ground to a fine powder under liquid nitrogen. Approximately 0.3 g of homogenized tissue was transferred to a 15 mL centrifuge tube. A 5 mL extraction solution (isopropanol/water/formic acid, 80:19:1, *v*/*v*/*v*) was added, followed by homogenization (2 min) and ultrasonication (1 h at 4 °C). The homogenate was centrifuged (10,000× *g*, 10 min, 4 °C), and the supernatant was collected. The pellet was re-extracted twice with 1 mL dichloromethane under low-temperature ultrasonication (30 min), followed by centrifugation (10,000× *g*, 10 min, 4 °C). Supernatants from all three extractions were combined, evaporated to the aqueous phase under a nitrogen stream at 25 °C, and reconstituted to a final volume of 1 mL with methanol. The extract was vortexed, diluted two-fold with methanol, filtered through a 0.22 μm membrane filter, and subjected to ultra-performance liquid chromatography–tandem mass spectrometry (UPLC-MS/MS) analysis.

### 4.4. Assays for Antioxidant Enzyme Activity and Proline Accumulation Analysis

Axillary bud samples were processed according to the manufacturer’s protocols. Antioxidant enzyme activities, including those of superoxide dismutase (SOD), peroxidase (POD), and catalase (CAT), along with proline (Pro) contents, were quantified using antioxidant enzyme assay kits (BC0095, BC0205, BC0295, and BC5165) (Solarbio Lifesciences, Beijing, China). All assays were performed in triplicate with blank controls.

### 4.5. RNA Extraction, RNA Sequencing, and qRT-PCR

Extraction of total RNA was performed by using a TIANGEN kit (TIANGEN Biotech, Beijing, China) according to the manufacturer’s instructions. Briefly, RNA-seq libraries made from total RNA attached with poly-T oligo were prepared as described previously and then sequenced on the Illumina novoseq6000 platform [21]. All experiments were carried out with three biological replicates. RNA-seq analysis was carried out on passion fruit samples to investigate their transcriptomic alterations. The clean reads were aligned to the passion fruit genome (PRJCA004251) [22] using HISAT2 (v2.1.0), with gene expression quantified by FeatureCounts (v2.0.1) and normalized as Fragments Per Kilobase of exon model per Million mapped fragments (FPKM). KEGG pathway enrichment was performed using the R package cluster Profiler (v4.4.4) with FDR < 0.05. Real-time quantitative PCR (qRT-PCR) was performed according to a previous study [23]. The primer sequences used for qRT-PCR in this study are shown in Table S5.

### 4.6. Statistical Analyses

All three biological replicates were collated using Microsoft Excel 2014, and statistical analyses were performed using GraphPad Prism 10 (Graphpad, San Diego, CA, USA) and IBM SPSS Statistics (Version 25); the results are expressed as the mean ± standard error (Mean ± SD). ANOVA with Tukey’s HSD was applied to determine these groupings. Letters were typically placed above the bars or error bars to facilitate visual comparison. Groups labeled with the same letter did not differ significantly (*p* > 0.05), whereas those with different letters exhibited statistically significant differences (*p* < 0.05). Principal component analysis (PCA) implemented with R package stats v4.2.1, and hierarchical cluster analysis (HCA) performed using the R package pheatmap v1.0.12.

## 5. Conclusions

Our integrated physiological, transcriptomic, and hormonal analyses demonstrated that 50% partial leaf retention optimally promotes Passiflora edulis axillary bud outgrowth through coordinated auxin–cytokinin crosstalk. This treatment simultaneously upregulates auxin transport genes (*AUX/LAX-ARF*) and cytokinin activation genes (*LOG1*, *ARR1*) while modulating cell wall dynamics via *SAUR*-mediated expansion (*SAUR21*, *SAUR78*) and suppressing dormancy pathways (DRM1/APR). These findings provide mechanistic insights into tropical fruit bud regulation by demonstrating how modulated leaf retention orchestrates the crosstalk between phytohormone signaling and metabolic reprogramming during axillary activation. The 50% PLR strategy offers nurseries an immediately implementable technique to improve grafting efficiency, with potential applicability to other perennial crops where precise source–sink modulation is required for successful propagation.

## Data Availability

The original contributions presented in the study are included in the article/Supplementary Materials, further inquiries can be directed to the corresponding author.

## Supplementary Materials

The following supporting information can be downloaded at: https://www.mdpi.com/article/10.3390/plants14162483/s1, Supplementary Figure S1. Comparative analysis of differentially expressed genes in 0_vs_1/2 comparison. Supplementary Table S1. RNA-seq analysis data. Supplementary Table S2. List of significant enriched categories of GO annotation. Supplementary Table S3. List of significant enriched categories of KEGG annotation. Supplementary Table S4. List of DEGs. Supplementary Table S5. List of primers.
