# Supplementary material for: Efficient Production of Vigorous Scions by Optimizing Leaf Retention in Passiflora edulis
Source: Plants (Basel). 2025 Aug 10;14(16):2483. doi: 10.3390/plants14162483 (PMC12389625; doi:10.3390/plants14162483)
Supplement: Supplementary file 1 [file plants-14-02483-s001.zip › Supplementary figures.pdf]

Supplementary Figures

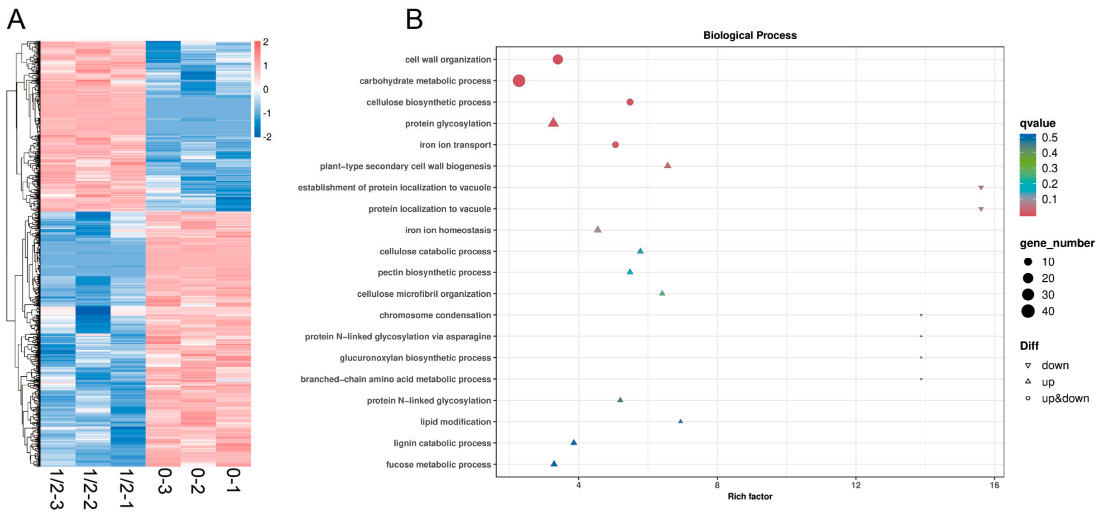

Figure S1. Comparative analysis of differentially expressed genes in 0_vs_1/2 comparison. (A) Hierarchical cluster analysis of DEGs in 0_vs_1/2 comparison group. (B) Enriched biological process GO terms in 0_vs_1/2 comparison group.
